# Impact of racism and discrimination on the physical and mental health among Aboriginal and Torres Strait Islander peoples living in Australia: a protocol for a scoping review

**DOI:** 10.1186/s13643-020-01480-w

**Published:** 2020-09-28

**Authors:** Camila A. Kairuz, Lisa M. Casanelia, Keziah Bennett-Brook, Julieann Coombes, Uday Narayan Yadav

**Affiliations:** 1 Department of Public Health, Torrens University, Sydney, Australia; 2 The George Institute for Global Health, Sydney, Australia; 3 Centre for Primary Health Care and Equity, Faculty of Medicine, UNSW, Sydney, Australia; 4 School of Population Health, UNSW, Sydney, Australia; 5 Forum for Health Research and Development, Dharan, Nepal

**Keywords:** Aboriginal, Mental health, Physical health, Racial discrimination, Racism, Scoping review, Torres Strait Islanders, Wellbeing

## Abstract

**Background:**

Racism is increasingly recognised internationally as a key factor contributing to health disparities. A comprehensive body of strong research from international authors has reported negative associations between racism and health outcomes. In Australia, although the literature is more limited, available findings follow global trends. Australia has an identified health gap between Aboriginal and Torres Strait Islander peoples and non-indigenous Australians, and despite efforts to bridge this gap, health inequities continue to exist. This scoping review aims to assess, analyse and synthesise the relationship between racism and discrimination on the physical and mental health of Aboriginal and Torres Strait Islander peoples living in Australia.

**Method:**

This is the study protocol for a scoping review. A systematic search will be conducted using five electronic databases: PubMed, CINAHL, Embase, Web of Science and the Australia’s National Institute for Aboriginal and Torres Strait Islander Health Research. The database search will include studies published between 2000 and 2020. Reference lists of the included articles will be searched. Outcome measures will include physical and mental health components including chronic conditions, depression and anxiety, psychological distress, social and emotional difficulties, suicide and health-related outcomes such as wellbeing and life satisfaction. Duplications will be removed, and titles and abstracts will be reviewed to select studies. Full-text screening of preselected studies will be performed by four reviewers independently, to select studies according to inclusion criteria. Included studies will be appraised for quality using appropriate tools tailored for each study design. Data will be extracted, and study findings and characteristics synthesised in a narrative summary.

**Discussion:**

Our scoping review will synthesise the evidence on the impacts of racism and discrimination in relation to the physical and mental health of Aboriginal and Torres Strait Islander peoples living in Australia. These findings could guide future health interventions by addressing the exposure of racism and racial discrimination in order to reduce health disparity. It is anticipated the findings to be of interest to policymakers, researchers, Aboriginal and Torres Strait Islander communities and community health organisations and other stakeholders interested in optimising public health interventions for and in partnership with Aboriginal and Strait Torres Islander communities of Australia.

****Scoping review registration**:**

The protocol for this review has been registered on the international prospective register of systematic reviews (PROSPERO). The registration ID is CRD42020186193.

## Background

International literature acknowledges racism and discrimination as a significant causal factor contributing to detrimental impacts on health [1]. Discrimination could be defined as the unequal and unjust treatment of individuals due to particular identifying characteristics such as race, ethnicity or gender [2]. According to Berman and Paradies (2010), racism is defined as the expression of racist beliefs, racist emotions or racist behaviours and practices that maintain or exacerbate inequality of opportunity among an ethnic-racial group. Research suggests racism has direct and indirect impacts on health. Racism can impact indirectly through reduced access to employment, housing and education [3], and by shaping negative health-related behaviours such as smoking and alcohol consumption [4]. In addition, racism can have a direct impact physically through fluctuations in physiological activity that has the potential to lead to permanent alterations associated with disease states [5].

Racism against indigenous populations is consistently reported worldwide [6–9], and racism has been acknowledged as a key determinant of health inequity faced by indigenous peoples [10]. Data from a population study, including 23 countries, provides evidence that indigenous groups experience poorer health and social outcomes than their respective benchmark populations [11]. Based on those findings, the authors concluded by recommending the development of targeted policy responses to indigenous health. Furthermore, the “2030 Agenda for Sustainable Development” by the United Nations has clearly identified the importance of addressing indigenous inequalities by referring explicitly to indigenous people six times within the political declaration; the education and zero hunger goals and on the follow-up section calling for indigenous people’s participation [12]. In Australia, discrimination and racism towards Aboriginal and Torres Strait Islander peoples is a reality that is exacerbated by collective experiences and memory of abuse, dispossession and intergenerational trauma from colonisation [13]. According to the 2014–2015 National Aboriginal and Torres Strait Islander Social Survey, 33% of Aboriginal and Torres Strait Islander people aged 15 and over reported they had experienced unfair treatment in the previous 12 months because of their indigenous origins [14]. Moreover, a study from the state of Victoria found that 17% of Aboriginal and Torres Strait Islander adults had experienced at least one episode of racism in the last year compared with 4.5% of their non-indigenous counterparts [15]. A study conducted among young Aboriginal and Torres Strait Islander children showed a high prevalence of perceived racism from either adults or peers [16].

It has been recognised for many years the health disadvantage that Aboriginal and Torres Strait Islander peoples have in comparison to their non-indigenous counterparts due to the ongoing colonialization of Australia [17]. For example, for Aboriginal and Torres Strait Islander people born in 2015–2017, the life expectancy was estimated to be 8.6 and 7.8 years lower than other Australian male and female counterparts, respectively [18]. Moreover, Aboriginal and Torres Strait Islander people experienced a total disease burden (disability-adjusted life years or DALYs) that was 2.3 times that of other Australian populations in 2011 [19] and are nearly three times more likely to be physiologically distressed and two times more likely to die by suicide than other Australians [20]. The government and the public health sector refer to these disparities as “The gap”. In the year 2008, the prime minister Kevin Rudd signed a statement of intent between the Government of Australia and Aboriginal and Torres Strait Islander peoples referred to as the “close the gap initiative” which was intended to achieve equality of health status and life expectancy between Aboriginal and Torres Islander peoples and other Australian populations [21]. Despite these efforts, 10 years after the commencement of the “close the gap” strategy, the prime minister reported that negligible progress had been made in reducing the gap [22].

Failure of programmes to improve health inequities has been attributed to strategies that are directed more towards lifestyle factors whilst racism is rarely addressed in intervention design and delivery [23, 24]. In 2018, the Australian Medical Association highlighted the importance of addressing institutional and interpersonal racism as a new target to bridge the gap [25]. A recently published systematic review, including studies with different ethnic minorities in the USA, found strong evidence of the negative association between racism and health [3]. Similar to the international evidence, primary studies performed in Australia have found positive associations between racism and negative mental health effects on Aboriginal and Torres Strait Islanders [26–28]. The negative association with physical health has also been documented [16, 29]. However, in Australia, there is scarce of systematic overview on the impact of racism and discrimination on physical and mental health outcomes among Aboriginal and Torres Strait Islander peoples. Therefore, this scoping review aims to assess, analyse and synthesise the relationship between racism and discrimination on the physical and mental health of Aboriginal and Torres Strait Islanders.

## Methods

This scoping review will follow the reporting guidelines and criteria set in Preferred Reporting Items for Systematic Review (PRISMA) [30]. Additionally, completed PRISMA extension for scoping reviews (PRISMA-ScR) checklist demonstrating the recommended items to include in a scoping review protocol, along with the location of each item within the document [31], can be found in Additional file 1. The review will be performed within an eight months’ time frame (May to December 2020) and will follow the stages proposed in the framework by Arksey et al. [32] and Levac [33] for scoping reviews.

The search will be conducted in English and will include studies published between the 1st of January of 2000 and June 2020. The rationale behind the search from the year 2000 to 2020 is findings from a meta-analysis study which showed that searches including articles from the last 20 years did not result in the loss of any primary study [34].

### Eligibility criteria

This scoping review will include quantitative studies that have measured the association between racism and physical and mental health outcomes among Aboriginal and Torres Strait Islander peoples of Australia. Only studies using quantitative methods (control-case studies, cross-sectional and cohort studies) will be included. This review will include studies in the English language conducted among Australia with indigenous population.

We will exclude experimental studies (randomised controlled trials (RCTs) and controlled clinical trials (*CCTs*)), and studies found only as abstract and those published in other than the English language. Publications like letters to the editor, commentaries, editorials and reviews will be excluded from this study. Additionally, studies that reported chronic disease like autoimmune disorders, arthritis and cystic fibrosis will be excluded from this review.

### Participants

In Australia, there are two distinctly recognised groups of indigenous peoples: Aboriginal peoples and Torres Islander peoples. The term “indigenous” is used today to describe both Aboriginal and Torres Strait Islander peoples. However, it is important to acknowledge that each has its own established values and protocols and own unique ways of expressing them [35, 36]. Furthermore, many Aboriginal and Torres Strait Islander people do not like to be referred to as “indigenous” as the term is considered to generic [37]. This review will study health outcomes on Aboriginal and Torres Strait Islander peoples of Australia of all ages without distinction of gender or socio-demographic characteristics.

### Data sources, search strategy and study selection

We will conduct a systematic search using five electronic databases: PubMed, CINAHL, Embase, Web of Science and the Australia’s National Institute for Aboriginal and Torres Strait Islander Health Research. The search will be performed using combinations of different keywords related to “Aboriginal and Torres Islander peoples”, “Racism”, “Discrimination” and “health”, using “OR” and “AND” as outlined in the Box 1 below.

**BOX 1:** List of search terms

**#1.** Racism OR Discrimination OR “Racial Prejudice(s)” OR “Racial discrimination” OR “Covert racism(s)” OR Harass OR Bully OR “Unfair treat” OR Oppress. **AND** **#2.** “mental health” OR Depression OR Anxiety OR stress OR Distress OR Suicide OR “quality of life” OR “self-efficacy” OR “satisfaction with illness” OR “satisfaction with life” OR “Psychological distress” OR “emotional problems” OR “Psychological illness.” **OR** **#3.** “physical health” OR “wellbeing” OR “cancer” OR “cardiovascular disease” OR “blood pressure” OR “Hypertension” OR “dysfunctional breathing” OR “Respiratory difficulties” OR “Chronic Obstructive Pulmonary Disease” OR “disease” OR “Life satisfaction” OR “Quality of life” OR “BMI” OR “Body max index” OR “ Asthma” OR “Cardiovascular disease” OR “Blood pressure” OR “Hypertension” OR “Heart disease” OR “chronic conditions” OR “Chronic disease” OR smoking OR tobacco OR “Alcohol” OR Drug OR “Substance use.” **AND** **#4.** Indigenous OR “Indigenous people(s)” OR Aboriginal OR “Torres Strait Islander” OR “First people(S)” **AND** **#5.** Australia OR “Rural Australia” OR “Remote Australia” OR “Urban Australia”	

We will manually consult the bibliography of the review articles to find additional citations. A university librarian and experts working on Aboriginal and Torres Strait Islander Health Research were consulted to ensure the search strategies were appropriate and current.

The search results will be imported to Endnote, and duplicates will be deleted. Four reviewers will screen titles and abstracts independently to select eligible papers. Pre-selected papers will be full text assessed independently by the five reviewers according to inclusion criteria. Any discrepancies between reviewers during the screening process regarding inclusion criteria will be resolved by discussion and consensus. If consensus cannot be reached, another reviewer will be included to decide on inclusion. The rationale for study exclusion will be recorded as part of the screening process. The screening process will be documented as a PRISMA flow diagram.

### Exposure measures

The measurement of racism is complex and is not completely developed [38]. However, racism can occur at different levels [39]. Interpersonal racism can be defined as the hierarchical and socially consequential valuation of a racial group [40] and the unfair treatment of people on the basis of their race or ethnicity [2]. Systemic or institutional racism is expressed through policies and practices held by institutions that result in less benefits to the oppressed group [29].

For the purpose of this study, the exposure measure will be perceived as interpersonal or institutional racism understood as the perception of receiving an inequal valuation or unfair treatment for being an Aboriginal or Torres Strait Islander person in Australia. This includes self-reported racism and racism reported by a child’s carer or a witness such as family or friends. Studies with all exposure time frames will be included.

### Outcome measures

The most universally accepted definition of health is the one proposed by the World Health Organization (WHO) which states that health is *a state of complete physical, mental and social wellbeing and not merely the absence of disease or infirmity* [41]. The National Aboriginal Community Controlled Health Organisation adopted in 1979 the following definition of healt h[42]: *Aboriginal health does not mean the physical wellbeing of an individual, but refers to the social, emotional and cultural wellbeing of the whole community. For Aboriginal people, this is seen in terms of the whole life view.* It includes social justice, equity, rights, traditional knowledge, traditional healing and connection to a country [43] and includes mental, physical, cultural and spiritual health [44].

Following this definition of health and with the aim of studying health outcomes in an integral way, the outcomes measured will include both physical and mental health components along with other health-related outcomes. In this study, physical health outcomes will include chronic conditions such as cardiovascular disease, diabetes, chronic obstructive pulmonary disease (COPD) and asthma, hypertension, cancer, abnormal body mass index [44] and risky behaviours (for instance, smoking, alcohol consumption and other substance use). Mental health outcomes will include psychological factors like depression and anxiety, psychological distress, social and emotional wellbeing, illness representations (satisfaction with life, quality of life, self-efficacy, satisfaction with illness) and suicide [45].

### Quality assessment

The quality appraisal of the studies included in the review will be performed using the Joanna Briggs Institute critical appraisal tools, according to each study design [46]. Four authors will independently assess the risk of bias of the included studies and resolve disagreements by discussion to reach consensus or with a vote of majority if they fail to reach consensus. Study authors will be contacted for additional information about the studies and for clarification of any aspect of the study as required. The results of the risk assessment will be portrayed in tables, and the respective commentary about the elements that lead to the overall risk assessment and study judgement will be detailed through a systematic narrative description.

### Data extraction

One team member will extract data from the included articles, and another four members will randomly check 10% of the articles meeting eligibility and exclusion criteria. The data to be extracted will include author, year of publication, journal, type of study (study design), location of the study (state or city) sample size, sample demographic characteristics, exposure measure including tools or instruments, exposure timeframe and severity. It will also include the strength and direction of the associations between racism and health outcomes, along with the type of data used to quantify the association (odds ratio, hazard ratio, correlation coefficients). The data will be compared; any discrepancies will be resolved by consensus or with the help of a fifth reviewer.

### Data synthesis

The data of the studies included will be analysed following a descriptive synthesis process based on the recommendations of the Joanna Briggs Institute Reviewer’s Manual [47]. Two members of this review team who represent Aboriginal and Torres Strait Islander people living in Australia will validate our interpretations of the data.

## Discussion

Despite the strong international evidence of the negative impact of racism on the health outcomes of different ethnic groups [3], public health interventions have not adequately addressed the racism problems faced by Aboriginal and Torres Strait Islander peoples of Australi a[24]. Evidence has shown that the lack of tailored interventions towards racism, and discrimination is an important factor contributing to the failure of the programme efforts [24, 25] and it could be the same for a programme aimed to improve physical and mental health outcome.

Evidence from other countries shows that initiatives to address racism have the potential to improve health [48]. However, advancing the creation of effective interventions requires the existence of solid evidence upon the unique characteristics of Aboriginal and Torres Strait Islander peoples [49]. This highlights the imperative need for research to guide this important work.

This study will be the first scoping review to map the magnitude of racism and its association with a diverse set of physical and mental outcomes among Aboriginal and Torres Strait Islander peoples. The results of this review will provide the explicit picture of racism, discrimination and its impact on the physical and mental health of Aboriginal and Torres Strait Islander peoples. This will guide the researchers, policymakers and the change-makers to design the public health interventions to combat this important social issue and thereby reducing the persistent disparities between Aboriginal and Torres Strait Islander peoples and other Australian populations. Additionally, our study will also add evidence on how racism and discrimination have been associated with risky health behaviours (psychoactive substance use) or poor lifestyle behaviours within the context of intergenerational trauma and ongoing impacts of colonisation. Furthermore, understanding the available information and the design limitations of current studies may uncover knowledge gaps on the topic and can guide future interventions aimed to improve the physical and mental health of Aboriginal and Torres Strait Islander people and communities.

## Supplementary information

**Additional file 1.**

## Data Availability

Not available

## Abbreviations

DALY: Disability-adjusted life years
PRISMA: Preferred Reporting Items for Systematic Reviews
PRISMA-ScR: PRISMA extension for scoping reviews
WHO: World Health Organization
